# Atrial fibrillation: trends in prevalence and antithrombotic prescriptions in the community

**DOI:** 10.1007/s12471-022-01667-x

**Published:** 2022-03-01

**Authors:** L. P. T. Joosten, A. R. de Boer, E. J. B. van Eerde, S. van Doorn, A. W. Hoes, M. L. Bots, F. H. Rutten, G. J. Geersing

**Affiliations:** 1 Julius Center for Health Sciences and Primary Care, University Medical Center Utrecht, Utrecht University, Utrecht, The Netherlands; 2 Dutch Heart Foundation, The Hague, The Netherlands

**Keywords:** Atrial fibrillation, Prevalence, Antithrombotic treatment, VKA, NOAC, Primary care

## Abstract

**Introduction:**

In the past decade, the atrial fibrillation (AF) landscape, including the treatment modalities, has drastically changed. This raises the question how AF prevalence and choices in antithrombotic therapy prescription have developed in the community over time.

**Methods:**

Routine care data from the Julius General Practitioners’ Network (JGPN) were used to calculate the yearly prevalence of AF and to quantify the percentage of all patients who were prescribed a platelet inhibitor, vitamin K antagonist (VKA), non-VKA oral anticoagulant (NOAC) or no antithrombotic medication. To explore whether certain patient characteristics are associated with selective prescription of oral anticoagulants (OAC), we applied logistic regression analyses.

**Results:**

From 2008 through 2017, the JGPN database included 7459 unique AF patients. During this period, the prevalence of AF increased from 0.4% to 1.4%. The percentage of patients prescribed a VKA declined from 47% to 41%, whereas the percentage of patients prescribed a NOAC rose from 0% to 20%. In patients with new-onset AF, older age, heart failure, diabetes mellitus, vascular disease and dementia were independently associated with a higher likelihood of VKA rather than NOAC prescription. In 2017, 25% of all patients with AF and a CHA_2_DS_2_-VASc score ≥ 2 were not prescribed OAC therapy (i.e. 8% with platelet inhibitor monotherapy and 17% without any antithrombotic therapy).

**Conclusion:**

Between 2008 and 2017, AF prevalence in the community more than tripled. Prescription patterns showed possible ‘channelling’ of VKAs over NOACs in frailer, elderly patients, whereas still about one in every four AF patients with a CHA_2_DS_2_-VASc score ≥ 2 was not prescribed any prophylactic OAC therapy.

**Supplementary Information:**

The online version of this article (10.1007/s12471-022-01667-x) contains supplementary material, which is available to authorized users.

## What’s new?

- The prevalence of reported atrial fibrillation (AF) in the general population more than tripled, from 0.4% in 2008 to 1.4% in 2017.
- In patients with new-onset AF, older age and concurrent presence of heart failure, diabetes, vascular disease and dementia were independently associated with a higher likelihood of vitamin K antagonist (VKA) rather than non-VKA oral anticoagulant prescription.
- In 2017, approximately one in every four patients with a diagnosis of AF and a CHA_2_DS_2_-VASc score ≥ 2 did not receive prophylactic oral anticoagulant therapy.

## Introduction

Atrial fibrillation (AF) is the most common cardiac arrhythmia among adults. AF patients are at greater risk of stroke and thromboembolism than patients without AF. On average, the stroke and thromboembolic risk in patients with AF is 2–3% per year, but this can be as high as 14% per year in untreated AF patients with multimorbidity, as summarised by the CHA_2_DS_2_-VASc risk model [1]. If the CHA_2_DS_2_-VASc score is equal to or exceeds 2 points, the stroke risk is considered high enough to warrant chronic oral anticoagulant (OAC) therapy for stroke prevention [1, 2]. Still, there is uncertainty about this threshold [3, 4].

Such prophylactic OAC therapy can be categorised into vitamin K antagonists (VKAs) and non-VKA OACs (NOACs). Although both VKAs and NOACs are effective in preventing stroke, they inherently also increase the bleeding risk [5–11]. Patients prescribed NOACs have a lower risk of intracranial bleeding compared with those taking VKAs, but a higher risk of gastrointestinal haemorrhage (particularly in the elderly) [11]. Platelet inhibitors are no longer indicated for stroke prevention in AF [2], because they are far less effective in stroke risk reduction than OAC therapy (22% vs 64%), and they are (nearly) not effective at all in those over 75 years [12, 13]. Nevertheless, platelet inhibitors are sometimes prescribed, notably in patients with (presumable) contraindications for VKAs or NOACs or in patients unwilling to receive OAC therapy.

In this changing AF landscape with changing treatment modalities, the question is how AF prevalence and the choices in prescription of OACs have developed over time. Therefore, the aim of this study was to describe trends in AF prevalence and patterns of antithrombotic therapy prescriptions in the community. Furthermore, we explored if certain patient characteristics are associated with selective OAC prescription (i.e. channelling).

## Methods

Data from the Julius General Practitioners’ Network (JGPN) were used for this study. The JGPN database contains pseudo-anonymous routine healthcare data from structured fields in electronic medical records of a large, ongoing, dynamic cohort consisting of all patients of the approximately 160 affiliated general practitioners (GPs) in the city of Utrecht and its vicinity in the Netherlands. The JGPN population is representative of the Dutch community with regard to sex and age and consisted of approximately 385,000 patients in 2017 [14].

### Data extraction

Patients with AF were identified in the JGPN database by using the International Classification of Primary Care (ICPC) code K78 (AF or atrial flutter), from 1 January 2008 to 31 December 2017 [15]. The following variables were extracted: sex, age, medical history using ICPC codes (see Table S1 in Electronic Supplementary Material) and cardiovascular medication prescriptions (see Table S2 in Electronic Supplementary Material). Medication prescriptions were classified according to the Anatomical Therapeutic Chemical classification system. Antithrombotic therapy was divided into three categories: VKA, NOAC and platelet inhibitor therapy. Medication prescription was not necessarily initiated by the GP but may have been started by a hospital specialist and continued by the GP.

### Statistical analysis

Baseline characteristics of AF patients are reported for 2008 (if AF was first recorded in or before 2008) or for the year AF was first recorded (if this was after 2008 and before 2018). They are presented as count and percentage for categorical variables and as median with interquartile range (IQR) for continuous variables.

The prevalence of reported AF was calculated for each year of the entire study period, whereby the whole JGPN population was placed in the denominator. In addition, the prevalence of AF was stratified by sex and by age (< 55 years, 55–64 years, 65–74 years, 75–84 years and ≥ 85 years).

The percentages of all AF patients who were prescribed VKA monotherapy, NOAC monotherapy, platelet inhibitor monotherapy, a combination of these antithrombotic treatments or no antithrombotic medication were calculated for each year of the entire study period. In addition, for the group of patients with a diagnostic code for AF and with a CHA_2_DS_2_-VASc score ≥ 2, the percentage of patients who were not prescribed OAC therapy (i.e. platelet inhibitor monotherapy or no antithrombotic therapy at all) was calculated for each year of the study period, to investigate possible changes over time in the percentage of patients who did not receive OAC therapy while this was considered necessary.

To explore the association between all predefined patient characteristics and VKA versus NOAC prescriptions in patients with new-onset AF, univariable logistic regression analyses were performed on the data of the year in which a new diagnostic code for AF or atrial flutter (ICPC code K78) for a certain patient was reported. To create a final set of variables that may be independently associated with VKA or NOAC prescription in patients with new-onset AF, multivariable logistic regression analyses with stepwise backward elimination (eliminated if *p*-value ≥ 0.05) were applied. Only AF patients who were prescribed OACs (either VKAs or NOACs) were included in these analyses, since the indication for prophylactic antithrombotic therapy is overall the same for this patient group. The group of patients who were prescribed a combination of antithrombotic treatments consisted of patients who switched antithrombotic medication within the concerning year(s) and patients who truly received antithrombotic medications from different medication groups at the same time. Because it was not possible to distinguish between them in our dataset, patients within this group were excluded from the univariable and multivariable logistic regression analyses in the year(s) in which this combination therapy was recorded.

All statistical analyses were performed using IBM SPSS Statistics 25.0.0.2 [16].

## Results

From 1 January 2008 through 31 December 2017, the JGPN database included 7459 unique patients with ICPC code K78 (AF or atrial flutter). The median follow-up time was 4 years (IQR 2–7) (Tab. 1). The median age was 74 years (IQR 65–82), 51.4% were men, and the median CHA_2_DS_2_-VASc score was 3 (IQR 2–4). The most prevalent comorbidity registered was hypertension (49.8%). Of the cardiovascular medication, beta blockers (62.7%) were most often prescribed, alongside antithrombotic therapy (76.0%).

**Table 1:** Baseline characteristics of patients with atrial fibrillation

Variable	Patients (*N* = 7459)
Follow-up time, years	4 (2–7)
Male sex	3836 (51.4)
*Age, years*	74 (65–82)
< 55	698 (9.4)
55–64	1131 (15.2)
65–74	1993 (26.7)
75–84	2257 (30.3)
≥ 85	1380 (18.5)
CHA_2_DS_2_-VASc score	3 (2–4)
CHA_2_DS_2_-VASc score ≥ 2	5829 (78.1)
Heart failure	1250 (16.8)
Hypertension	3717 (49.8)
Diabetes mellitus	1497 (20.1)
CVA or TIA	833 (11.2)
Vascular disease^*a*^	1617 (21.7)
Renal impairment^*b*^	1313 (17.6)
Dementia	256 (3.4)
Asthma or COPD	1295 (17.4)
Malignancy^*c*^	623 (8.4)
History of bleeding^*d*^	1407 (18.9)
Antithrombotic therapy	5667 (76.0)
Beta blocker	4680 (62.7)
Calcium channel blocker	1543 (20.7)
*Digoxin*	1451 (19.5)

*CVA* cerebrovascular accident, *TIA* transient ischaemic attack,* COPD* chronic obstructive pulmonary disease

Data are median (interquartile range) or n (%)

^a^ Coronary artery disease (angina pectoris, acute myocardial infarction, other/chronic ischaemic heart disease) or peripheral vascular (arterial or venous) disease (intermittent claudication, thrombophlebitis/phlebothrombosis, deep vein thrombosis in pregnancy)

^b^ International Classification of Primary Care (ICPC) code U99.01 (renal impairment) or estimated glomerular filtration rate < 60 ml/min per 1.73 m^2^

^c^ Five most prevalent malignancies in the Netherlands (apart from skin cancer): breast cancer, prostate cancer, colon cancer, lung cancer and haematological cancer

^d^ Posttraumatic extradural/subdural/intracerebral haemorrhage, haemoptysis, epistaxis, haematemesis, melaena, haematochezia, haematuria, menorrhagia, postpartum haemorrhage

### Prevalence of atrial fibrillation

**Fig. 1:**
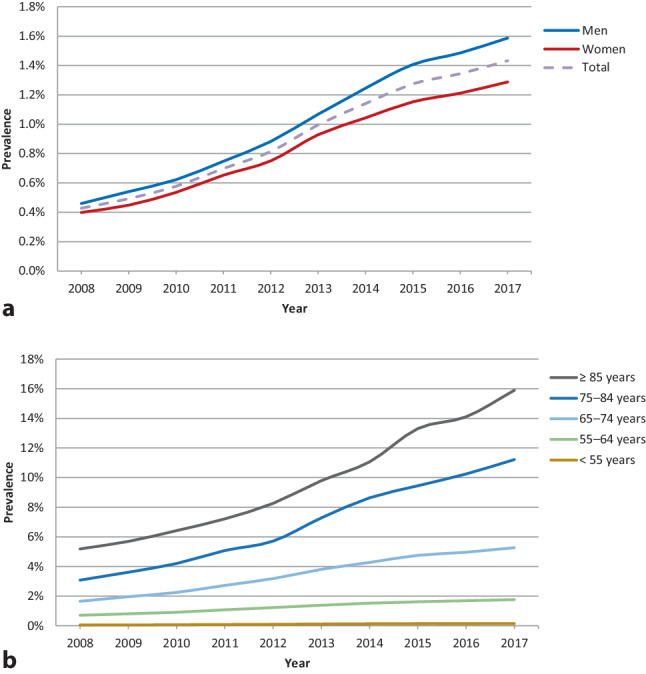
Trends in prevalence of atrial fibrillation in primary care, stratified by **a** sex and **b** age. (This figure was reprinted from: Joosten LPT, van Eerde EJB, Rutten FH, Geersing GJ. Ontwikkelingen in prevalentie van atriumfibrilleren en anti-trombotica voorschriften. In: De Boer AR, van Dis I, Visseren FLJ, Vaartjes I, Bots ML (eds). Hart- en vaatziekten in Nederland 2019, cijfers over incidentie, prevalentie, ziekte en sterfte. The Hague: Dutch Heart Foundation; 2019. Copyright, with permission from the Dutch Heart Foundation.)

Prevalence of reported AF increased over time, from 0.43% (95% confidence interval (CI) 0.41%–0.45%) in 2008 to 1.43% (95% CI 1.39%–1.47%) in 2017 (Fig. 1). Men had a higher AF prevalence than women (1.6% vs 1.3% in 2017). AF prevalence was highest in the oldest patients (0.1% in patients < 55 years vs 15.9% in those aged ≥ 85 years in 2017) and, over time, the increase was more pronounced in the older age categories than in the younger age categories.

### Prescription of antithrombotic therapy

**Fig. 2:**
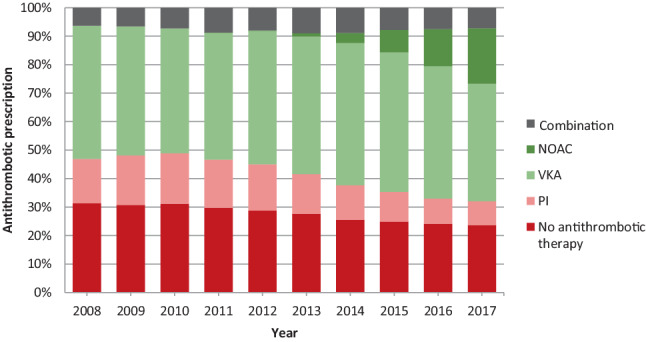
Trends in antithrombotic prescriptions in all patients with atrial fibrillation in primary care. *NOAC* non-vitamin K antagonist oral anticoagulant, *VKA* vitamin K antagonist,* PI* platelet inhibitor. (This figure was reprinted from: Joosten LPT, van Eerde EJB, Rutten FH, Geersing GJ. Ontwikkelingen in prevalentie van atriumfibrilleren en anti-trombotica voorschriften. In: De Boer AR, van Dis I, Visseren FLJ, Vaartjes I, Bots ML (eds). Hart- en vaatziekten in Nederland 2019, cijfers over incidentie, prevalentie, ziekte en sterfte. The Hague: Dutch Heart Foundation; 2019. Copyright, with permission from the Dutch Heart Foundation.)

During the entire study period, most patients were prescribed VKA monotherapy, with a decline from 46.7% in 2008 to 41.3% in 2017 (Fig. 2). The percentage of patients who were prescribed NOAC monotherapy steadily increased, from 0.0% in 2011 to 19.5% in 2017. Most of the 1608 AF patients who were prescribed NOAC monotherapy for the first time during the study period, were new-onset AF patients (57.4%). The percentage of patients with a diagnostic code for AF and with a CHA_2_DS_2_-VASc score ≥ 2 (justifying OAC therapy for stroke prevention) who were not prescribed OAC therapy decreased from 42.2% in 2008 (consisting of 15.3% who were prescribed platelet inhibitor monotherapy and 26.9% who were prescribed no antithrombotic therapy at all) to 25.4% in 2017 (8.5% were prescribed platelet inhibitor monotherapy and 16.9% were not prescribed any antithrombotic therapy).

### Selective anticoagulant prescription

In univariable logistic regression analyses, each pre-defined patient characteristic might be related to VKA prescription rather than NOAC prescription, except for sex, hypertension, asthma or COPD, history of cerebrovascular accident or transient ischaemic attack and history of bleeding (not statistically significant) and except for history of malignancy, which in itself might be related to NOAC prescription compared with VKA prescription (Tab. 2). After multivariable logistic regression analyses with stepwise backward elimination, older age and concurrent heart failure, diabetes mellitus, vascular disease and dementia were independently associated with a higher likelihood of VKA rather than NOAC prescription, whereas hypertension and malignancy were independently associated with a higher likelihood of NOAC rather than VKA prescription (Tab. 2). Dementia was most strongly associated with a higher likelihood of VKA rather than NOAC prescription (adjusted odds ratio 2.11, 95% CI 1.04–4.28 for patients with dementia compared with patients without dementia). Regarding age, for every year increase in age, the relative proportion of prevalent VKA prescriptions versus prevalent NOAC prescriptions seemed to increase with a factor 1.03 (95% CI 1.02–1.04).

**Table 2:** Patient characteristics probably associated with VKA versus NOAC prescription in patients with new-onset atrial fibrillation diagnosed from 2011 through 2017

**Univariable analyses**			
*Variable*	*VKA* *(N* *=* *1842)*	*NOAC* *(N* *=* *603)*	*Unadjusted odds ratio (95% CI)*
Male sex	939 (51.0)	305 (50.6)	1.02 (0.85–1.22)
Age, years	76 (69–83)	72 (65–79)	1.03 (1.03–1.04)
Age ≥ 75 years	1039 (56.4)	235 (39.0)	2.03 (1.68–2.45)
CHA_2_DS_2_-VASc score	3 (2–4)	3 (2–4)	1.21 (1.14–1.28)
CHA_2_DS_2_-VASc score ≥ 2	1570 (85.2)	467 (77.4)	1.68 (1.34–2.12)
Heart failure	365 (19.8)	60 (10.0)	2.24 (1.67–2.99)
Hypertension	934 (50.7)	316 (52.4)	0.93 (0.78–1.12)
Diabetes mellitus	439 (23.8)	102 (16.9)	1.54 (1.21–1.95)
CVA or TIA	160 (8.7)	48 (8.0)	1.10 (0.79–1.54)
Vascular disease^a^	349 (18.9)	76 (12.6)	1.62 (1.24–2.12)
Renal impairment^b^	444 (24.1)	99 (16.4)	1.62 (1.27–2.06)
Dementia	84 (4.6)	9 (1.5)	3.15 (1.58–6.31)
Asthma or COPD	313 (17.0)	110 (18.2)	0.92 (0.72–1.17)
Malignancy^c^	158 (8.6)	69 (11.4)	0.73 (0.54–0.98)
History of bleeding^d^	349 (18.9)	107 (17.7)	1.08 (0.85–1.38)
**Multivariable analyses**^e^			
*Variable*	*Adjusted odds ratio (95% CI)*		
Age, years	1.03 (1.02–1.04)		
Heart failure	1.72 (1.27–2.32)		
Hypertension	0.80 (0.66–0.97)		
Diabetes mellitus	1.45 (1.13–1.85)		
Vascular disease^a^	1.38 (1.05–1.82)		
Dementia	2.11 (1.04–4.28)		
Malignancy^c^	0.63 (0.46–0.85)		

*VKA* vitamin K antagonist, *NOAC* non**-**vitamin K antagonist oral anticoagulant, *CI* confidence interval, *CVA* cerebrovascular accident, *TIA* transient ischaemic attack,* COPD* chronic obstructive pulmonary disease

Data are n (%) or median (interquartile range)

^a^ Coronary artery disease (angina pectoris, acute myocardial infarction, other/chronic ischaemic heart disease) or peripheral vascular (arterial or venous) disease (intermittent claudication, thrombophlebitis/phlebothrombosis, deep vein thrombosis in pregnancy)

^b^ International Classification of Primary Care (ICPC) code U99.01 (renal impairment) or estimated glomerular filtration rate < 60 ml/min per 1.73 m^2^

^c^ Five most prevalent malignancies in the Netherlands (apart from skin cancer): breast cancer, prostate cancer, colon cancer, lung cancer and haematological cancer

^d^ Posttraumatic extradural/subdural/intracerebral haemorrhage, haemoptysis, epistaxis, haematemesis, melaena, haematochezia, haematuria, menorrhagia, postpartum haemorrhage

^e^ Multivariable analyses with stepwise backward elimination (eliminated if p-value ≥ 0.05) and with age and CHA_2_DS_2_-VASc score as continuous instead of dichotomous variables

## Discussion

This study was conducted in the general population to investigate trends in AF prevalence and antithrombotic treatment prescriptions from 2008 through 2017.

### Comparison with literature

The prevalence of reported AF more than tripled in our study (from 0.4% in 2008 to 1.4% in 2017). Krijthe et al. estimated that the prevalence will more than double from 2010 to 2060 [17]. Interestingly, our study indicates that, at least in the Netherlands, the steep increase in AF prevalence occurs in a much shorter time period (i.e. tripling in a decade instead of doubling in half a century). Although the purpose of our study was not to explain the observed trends, this steeper than expected increase in reported AF prevalence deserves some consideration.

Firstly, several factors may have contributed to the steep increase in reported AF prevalence: (1) increased awareness of AF related to the introduction of NOACs and the updated Dutch and European AF guidelines; (2) recent developments in Dutch primary care, which include disease managing programmes for patients with increased cardiovascular risk; and (3) enhanced digitalisation, resulting in improved accurateness and completeness of (AF) registration in electronic healthcare records.

Secondly, in developed countries, a plausible reason for the steep increase in reported AF prevalence is a better survival after a first cardiovascular event, due to improved healthcare and an overall improvement in cardiovascular risk factor predisposition. This improved survival could expose patients to the spectrum of later-onset chronic cardiovascular disease, such as AF. This hypothesis is strengthened by studies in which a clear reduction in total cardiovascular morbidity and mortality over the last decades and a shift in the burden of cardiovascular morbidity from acute to chronic cardiovascular diseases, including the development of AF, were observed [18, 19].

In our study, 25.4% of all AF patients with a CHA_2_DS_2_-VASc score ≥ 2 were not prescribed OAC therapy (8.5% were prescribed platelet inhibitor monotherapy and 16.9% were not prescribed any antithrombotic therapy) in 2017. This is comparable to the results of the international GLORIA-AF registry (study period 2011–2014): 16.7% of new-onset AF patients with a CHA_2_DS_2_-VASc score ≥ 2 did not receive OAC therapy (10.0% were prescribed a platelet inhibitor and 6.7% were not prescribed any antithrombotic therapy) [20]. In the international GARFIELD-AF registry (study period 2009–2016), 38.0% of new-onset AF patients with an indication for OAC therapy did not receive any anticoagulation [21]. Since the percentages of undertreatment cannot be fully explained by patients with a contraindication for anticoagulants (around 2.2%) [22], all three studies (GLORIA-AF registry [20], GARFIELD-AF registry [21] and our study) clearly emphasise that antithrombotic treatment in AF patients still leaves room for improvement and undertreatment remains a point of attention for both patients and physicians [23, 24].

Identifying subgroups at risk of stroke due to inappropriate treatment should be the focus of new research. However, as a first step, we performed additional descriptive analyses, stratified by CHA_2_DS_2_-VASc score, to explore the characteristics of all AF patients who were prescribed a platelet inhibitor or no antithrombotic therapy at all in 2017 (see Table S3 in Electronic Supplementary Material). It seemed, among other things, that physicians regard platelet inhibitors as a reasonable alternative for OAC therapy or they do not consider initiating OAC therapy in AF patients with pre-existing vascular disease, such as coronary artery disease or peripheral vascular disease, perhaps because these patients are already prescribed a platelet inhibitor.

### Strengths and limitations

A major strength of our study is that we used uniformly registered, routine clinical practice data on trends in AF in primary care spanning a decade.

Two limitations, which are inherent to using data derived from structured fields in electronic health records, are: (1) lack of specific granular information (e.g. no differentiation based on AF subtype (paroxysmal, persistent, permanent) and inability to differentiate between primary versus secondary AF and between AF versus atrial flutter); and (2) risks of misclassification in predictor values used in the CHA_2_DS_2_-VASc model, misclassification in diagnosis and—to a lesser extent when using data from the JGPN database—misclassification in treatment. However, the JGPN consists of a dedicated group of GPs who have been trained in accurately coding diseases using ICPC codes. Moreover, Van Doorn et al. have demonstrated that the risk of substantial misclassification in individual predictors of the CHA_2_DS_2_-VASc model is relatively small in multivariable analyses, albeit present [25].

### Clinical implications

The clinical implications of this study are multiple. Firstly, the large increase in reported AF prevalence over time was far greater than previously expected [17]. This can lead to an increase in AF care, in particular care aimed at stroke prevention, which could, for example, be realised to a large extent through integrated management of AF in primary care [26].

Secondly, there is still room for improvement in stroke prevention by further reducing OAC undertreatment (i.e. platelet inhibitor monotherapy or no antithrombotic therapy at all) in patients with a CHA_2_DS_2_-VASc score ≥ 2.

Finally, the number of NOAC prescriptions is expected to increase further. We observed that the diminishing group of patients who were (still) prescribed a VKA for new-onset AF, were older and had more comorbidity (e.g. heart failure, diabetes mellitus and vascular disease, as has also been shown by the GARFIELD-AF registry [21]) than patients receiving a NOAC. Moreover, based on additional explanatory analyses over time we performed, we concluded that channelling of VKAs over NOACs in older patients and in patients with more comorbidity still took place in 2017, which was the first year in which more new-onset AF patients received a NOAC instead of a VKA (see Table S4 in Electronic Supplementary Material). In the Netherlands, GP guidelines on AF recommend to be cautious when prescribing a NOAC to these (aged) frail patients [27]. Although observational data suggest that certain NOACs are as safe as (or safer than) VKAs in frail elderly [28], more research is needed to confirm or refute the current caution in guidelines for this patient group. One such study is already on its way: the randomised controlled FRAIL-AF trial, in which frail AF patients on VKA therapy are switched to a NOAC [29]. Nonetheless, it is imaginable that the organisation of care for (frailer) VKA users—in the Netherlands, this is currently provided by the Dutch Thrombosis Services—may have to change in order to guarantee quality and continuity for AF patients who continue to take a VKA, for example by means of integrated management of AF in primary care [26].

## Conclusion

Between 2008 and 2017, the prevalence of reported AF in the community more than tripled. Prescription patterns of antithrombotic treatment showed possible channelling of VKAs over NOACs in frailer, elderly patients, whereas still about one in every four patients with a diagnostic code for AF and a CHA_2_DS_2_-VASc score ≥ 2 was not prescribed any prophylactic OAC therapy.

## Supplementary Information

**Table S1** International Classification of Primary Care (ICPC) codes used in this study

**Table S2** Anatomical Therapeutic Chemical (ATC) classification system codes used in this study

**Table S3** Characteristics of all patients with atrial fibrillation who were prescribed anticoagulation therapy, a platelet inhibitor or no antithrombotic therapy at all in 2017 (stratified by CHA_2_DS_2_-VASc score)

**Table S4** Patient characteristics probably associated with a VKA versus a NOAC prescription in patients with new-onset atrial fibrillation diagnosed in 2015, 2016 and 2017
