# Supplementary material for: Atrial fibrillation: trends in prevalence and antithrombotic prescriptions in the community
Source: Neth Heart J. 2022 Mar 1;30(10):459–65. doi: 10.1007/s12471-022-01667-x (PMC9475006; doi:10.1007/s12471-022-01667-x)
Supplement: Supplementary file 1 — Table S1 International Classification of Primary Care (ICPC) codes used in this study [file 12471_2022_1667_MOESM1_ESM.docx]

**Table S1** International Classification of Primary Care (ICPC) codes used in the study

| **Medical history** | **ICPC code** |
| --- | --- |
| Heart failure | K77 |
| Hypertension | K85, K86, K87 |
| Diabetes mellitus | T90 |
| CVA or TIA | K89, K90 |
| Vascular disease^a^ | K74, K75, K76, K92.01, K94, W77.03 |
| Renal impairment | U99.01 or eGFR < 60 ml/min per 1.73 m^2^ |
| Dementia | P70 |
| Asthma or COPD | R95, R96 |
| Malignancy**^b^** | B72, B73, B74, D75, R84, R85, X76, Y77 |
| History of bleeding**^c^** | A10, D14, D15, D16, N80.01, N80.02, N80.03, R06, R24, U06, W17, X06 |

^a^ Coronary artery disease (angina pectoris, acute myocardial infarction, other/chronic ischaemic heart disease) or
 peripheral vascular (arterial or venous) disease (intermittent claudication, thrombophlebitis/phlebothrombosis,
 deep vein thrombosis in pregnancy)
^b^ Five most prevalent malignancies in the Netherlands (apart from skin cancer): breast cancer, prostate cancer,
 colon cancer, lung cancer and haematological cancer
^c^ Posttraumatic extradural/subdural/intracerebral haemorrhage, haemoptysis, epistaxis, haematemesis, melena,
 haematochezia, haematuria, menorrhagia, postpartum haemorrhage

*ICPC* International Classification of Primary Care, *CVA* cerebrovascular accident, *TIA* transient ischemic attack, *eGFR* estimated glomerular filtration rate, *COPD* chronic obstructive pulmonary disease
