# Supplementary material for: Atrial fibrillation: trends in prevalence and antithrombotic prescriptions in the community
Source: Neth Heart J. 2022 Mar 1;30(10):459–65. doi: 10.1007/s12471-022-01667-x (PMC9475006; doi:10.1007/s12471-022-01667-x)
Supplement: Supplementary file 2 — Table S2 Anatomical Therapeutic Chemical (ATC) classification system codes used in this study [file 12471_2022_1667_MOESM2_ESM.docx]

**Table S2** Anatomical Therapeutic Chemical (ATC) classification system codes used in this study

| **Antithrombotic therapy** | **ACT code** |
| --- | --- |
| **Platelet inhibitor (PI)** | |
| Clopidogrel | B01AC04 |
| Acetylsalicylic acid | B01AC06 |
| Dipyridamole | B01AC07 |
| Carbasalate calcium | B01AC08 |
| Epoprostenol | B01AC09 |
| Prasugrel | B01AC22 |
| Ticagrelor | B01AC24 |
| Selexipag | B01AC27 |
| Combinations | B01AC30 |
| **Vitamin K antagonist (VKA)** | |
| Warfarin | B01AA03 |
| Phenprocoumon | B01AA04 |
| Acenocoumarol | B01AA07 |
| **Non-vitamin K antagonist oral anticoagulant (NOAC)** | |
| Dabigatran | B01AE07 |
| Rivaroxaban | B01AF01 |
| Apixaban | B01AF02 |
| Edoxaban | B01AF03 |
| **Other medication** | **ACT code** |
| Beta blocker | C07 |
| Calcium channel blocker | C08 |
| Digoxin | C01AA |
