# Supplementary material for: Atrial fibrillation: trends in prevalence and antithrombotic prescriptions in the community
Source: Neth Heart J. 2022 Mar 1;30(10):459–65. doi: 10.1007/s12471-022-01667-x (PMC9475006; doi:10.1007/s12471-022-01667-x)
Supplement: Supplementary file 3 — Table S3 Characteristics of all patients with atrial fibrillation who were prescribed anticoagulation therapy, a platelet inhibitor or no antithrombotic therapy at all in 2017 (stratified by CHA2DS2-VASc score) [file 12471_2022_1667_MOESM3_ESM.docx]

**Table S3** Characteristics of all patients with atrial fibrillation who were prescribed anticoagulation therapy, a platelet inhibitor or no antithrombotic therapy at all in 2017 (stratified by CHA_2_DS_2_-VASc score)

| **Variable** | **CHA_2_DS_2_-VASc <2** | | | **CHA_2_DS_2_-VASc ≥2** | | |
| --- | --- | --- | --- | --- | --- | --- |
|  | **ACT**  **(*N*= 333)** | **PI (*N*= 75)** | **No ATT (*N*= 539)** | **ACT**  **(*N*= 3014)** | **PI**  **(*N*= 385)** | **No ATT**  **(*N*= 766)** |
| Male sex | 299(89.8) | 63(84.0) | 422(78.3) | 1422 (47.2) | 193 (50.1) | 335 (43.7) |
| Age, years | 62(56-68) | 60(55-67) | 55(46-62) | 78.5(72-84) | 78(70.5-85) | 77(70-84) |
| Age ≥75 years | 0(0.0) | 0(0.0) | 0(0.0) | 1975 (65.5) | 238(61.8) | 458(59.8) |
| CHA_2_DS_2_-VASc score | 1 (0-1) | 1 (0-1) | 0 (0-1) | 4 (3-5) | 4 (3-5) | 3 (2-5) |
| Heart failure | 9 (2.7) | 0 (0.0) | 2 (0.4) | 766 (25.4) | 62 (16.1) | 176 (23.0) |
| Hypertension | 57(17.1) | 12(16.0) | 61(11.3) | 2022 (67.1) | 270(70.1) | 499(65.1) |
| Diabetes mellitus | 11 (3.3) | 1 (1.3) | 12 (2.2) | 855 (28.4) | 99 (25.7) | 184 (24.0) |
| CVA or TIA | 0 (0.0) | 0 (0.0) | 0 (0.0) | 523 (17.4) | 84 (21.8) | 96 (12.5) |
| Vascular disease^a^ | 9 (2.7) | 6 (8.0) | 5 (0.9) | 883 (29.3) | 173 (44.9) | 183 (23.9) |
| Renal impairment^b^ | 21 (6.3) | 3 (4.0) | 3 (0.6) | 902 (29.9) | 110 (28.6) | 185 (24.2) |
| Dementia | 1 (0.3) | 0 (0.0) | 0 (0.0) | 122 (4.0) | 15 (3.9) | 52 (6.8) |
| Asthma or COPD | 47 (14.1) | 9 (12.0) | 66 (12.2) | 659 (21.9) | 76 (19.7) | 169 (22.1) |
| Malignancy^c^ | 17 (5.1) | 8 (10.7) | 14 (2.6) | 410 (13.6) | 36 (9.4) | 90 (11.7) |
| History of bleeding^d^ | 85(25.5) | 17(22.7) | 73(13.5) | 1028 (34.1) | 114(29.6) | 215(28.1) |
| Beta blocker | 238(71.5) | 37(49.3) | 119(22.1) | 2127 (70.6) | 220(57.1) | 278(36.3) |
| Calcium channel blocker | 50 (15.0) | 5 (6.7) | 18 (3.3) | 790 (26.2) | 89 (23.1) | 107 (14.0) |
| Digoxin | 38 (11.4) | 2 (2.7) | 6 (1.1) | 581 (19.3) | 25 (6.5) | 43 (5.6) |

Data are n (%) or median (interquartile range)

^a^ Coronary artery disease (angina pectoris, acute myocardial infarction, other/chronic ischaemic heart disease) or peripheral vascular (arterial or venous) disease (intermittent claudication, thrombophlebitis/phlebothrombosis, deep vein thrombosis in pregnancy)

^b^ International Classification of Primary Care (ICPC) code U99.01 (renal impairment) or estimated glomerular filtration rate < 60 ml/min per 1.73 m^2^

^c^ Five most prevalent malignancies in the Netherlands (apart from skin cancer): breast cancer, prostate cancer, colon cancer, lung cancer and haematological cancer

^d^ Posttraumatic extradural/subdural/intracerebral haemorrhage, haemoptysis, epistaxis, haematemesis, melaena, haematochezia, haematuria, menorrhagia, postpartum haemorrhage

^e^ Multivariable analyses with stepwise backward elimination (eliminated if *p*-value ≥ 0.05) and with age and CHA_2_DS_2_-VASc score as continuous instead of dichotomous variables

*ACT* anticoagulation therapy (i.e. a vitamin K antagonist or a non-vitamin K antagonist oral anticoagulant), *PI* platelet inhibitor, *ATT* antithrombotic therapy, *CVA* cerebrovascular accident, *TIA* transient ischemic attack, *COPD* chronic obstructive pulmonary disease
