# Supplementary material for: Atrial fibrillation: trends in prevalence and antithrombotic prescriptions in the community
Source: Neth Heart J. 2022 Mar 1;30(10):459–65. doi: 10.1007/s12471-022-01667-x (PMC9475006; doi:10.1007/s12471-022-01667-x)
Supplement: Supplementary file 4 — Table S4 Patient characteristics probably associated with a VKA versus a NOAC prescription in patients with new-onset atrial fibrillation diagnosed in 2015, 2016 and 2017 [file 12471_2022_1667_MOESM4_ESM.docx]

**Table S4A** Patient characteristics probably associated with a VKA versus a NOAC prescription in patients with new-onset atrial fibrillation diagnosed in 2015

| **Univariable analyses** | | | |
| --- | --- | --- | --- |
| **Variable** | **VKA (*N*= 295)** | **NOAC (*N*= 115)** | **Unadjusted odds ratio (95% CI)** |
| Male sex | 150 (50.8) | 64 (55.7) | 0.82 (0.54-1.27) |
| Age, years | 77 (69-84) | 68 (59-76) | 1.07 (1.04-1.09) |
| Age ≥75 years | 171 (58.0) | 32 (27.8) | 3.58 (2.24-5.72) |
| CHA_2_DS_2_-VASc score | 3 (2-5) | 2 (1-3) | 1.53 (1.32-1.77) |
| CHA_2_DS_2_-VASc score ≥2 | 259 (87.8) | 76 (66.1) | 3.69 (2.20-6.21) |
| Heart failure | 71 (24.1) | 9 (7.8) | 3.73 (1.80-7.75) |
| Hypertension | 171 (58.0) | 47 (40.9) | 2.00 (1.29-3.09) |
| Diabetes mellitus | 72 (24.4) | 18 (15.7) | 1.74 (0.99-3.07) |
| CVA or TIA | 29 (9.8) | 8 (7.0) | 1.46 (0.65-3.29) |
| Vascular disease^a^ | 58 (19.7) | 14 (12.2) | 1.77 (0.94-3.31) |
| Renal impairment^b^ | 94 (31.9) | 11 (9.6) | 4.42 (2.27-8.62) |
| Dementia | 12 (4.1) | 4 (3.5) | 1.18 (0.37-3.73) |
| Asthma or COPD | 58 (19.7) | 17 (14.8) | 1.41 (0.78-2.54) |
| Malignancy^c^ | 41 (13.9) | 11 (9.6) | 1.53 (0.76-3.08) |
| History of bleeding^d^ | 66 (22.4) | 15 (13.0) | 1.92 (1.05-3.53) |
| **Multivariable analyses**^e^ | | | |
| **Variable** | **Adjusted odds ratio (95% CI)** | | |
| Age, years | 1.06 (1.04-1.08) | | |
| Renal impairment^b^ | 2.92 (1.46-5.84) | | |

Data are n (%) or median (interquartile range)

^a^ Coronary artery disease (angina pectoris, acute myocardial infarction, other/chronic ischaemic heart disease) or peripheral vascular (arterial or venous) disease (intermittent claudication, thrombophlebitis/phlebothrombosis, deep vein thrombosis in pregnancy)

^b^ International Classification of Primary Care (ICPC) code U99.01 (renal impairment) or estimated glomerular filtration rate < 60 ml/min per 1.73 m^2^

^c^ Five most prevalent malignancies in the Netherlands (apart from skin cancer): breast cancer, prostate cancer, colon cancer, lung cancer and haematological cancer

^d^ Posttraumatic extradural/subdural/intracerebral haemorrhage, haemoptysis, epistaxis, haematemesis, melaena, haematochezia, haematuria, menorrhagia, postpartum haemorrhage

^e^ Multivariable analyses with stepwise backward elimination (eliminated if *p*-value ≥ 0.05) and with age and CHA_2_DS_2_-VASc score as continuous instead of dichotomous variables

*VKA* vitamin K antagonist, *NOAC* non-vitamin K antagonist oral anticoagulant, *CI* confidence interval, *CVA* cerebrovascular accident, *TIA* transient ischemic attack, *COPD* chronic obstructive pulmonary disease

**Table S4B** Patient characteristics probably associated with a VKA versus a NOAC prescription in patients with new-onset atrial fibrillation diagnosed in 2016

| **Univariable analyses** | | | |
| --- | --- | --- | --- |
| **Variable** | **VKA (*N*= 221)** | **NOAC (*N*= 156)** | **Unadjusted  odds ratio (95% CI)** |
| Male sex | 119 (53.8) | 76 (48.7) | 1.23 (0.82-1.85) |
| Age, years | 78 (72-84.5) | 72 (66.25-79) | 1.07 (1.04-1.09) |
| Age ≥75 years | 144 (65.2) | 62 (39.7) | 2.84 (1.86-4.33) |
| CHA_2_DS_2_-VASc score | 4 (2.5-4) | 3 (2-4) | 1.35 (1.18-1.56) |
| CHA_2_DS_2_-VASc score ≥2 | 201 (91.0) | 121 (77.6) | 2.91 (1.61-5.27) |
| Heart failure | 50 (22.6) | 14 (9.0) | 2.97 (1.58-5.59) |
| Hypertension | 122 (55.2) | 82 (52.6) | 1.11 (0.74-1.68) |
| Diabetes mellitus | 56 (25.3) | 23 (14.7) | 1.96 (1.15-3.36) |
| CVA or TIA | 24 (10.9) | 13 (8.3) | 1.34 (0.66-2.72) |
| Vascular disease^a^ | 43 (19.5) | 16 (10.3) | 2.11 (1.14-3.91) |
| Renal impairment^b^ | 63 (28.5) | 36 (23.1) | 1.33 (0.83-2.13) |
| Dementia | 11 (5.0) | 0 (0.0) | - |
| Asthma or COPD | 37 (16.7) | 29 (18.6) | 0.88 (0.52-1.51) |
| Malignancy^c^ | 25 (11.3) | 13 (8.3) | 1.40 (0.69-2.84) |
| History of bleeding^d^ | 58 (26.2) | 30 (19.2) | 1.49 (0.91-2.46) |
| **Multivariable analyses** | | | |
| **Variable** | **Adjusted odds ratio (95% CI)** | | |
| Age, years | 1.06 (1.04-1.09) | | |
| Diabetes mellitus | 1.85 (1.06-3.22) | | |

Data are n (%) or median (interquartile range)

^a^ Coronary artery disease (angina pectoris, acute myocardial infarction, other/chronic ischaemic heart disease) or peripheral vascular (arterial or venous) disease (intermittent claudication, thrombophlebitis/phlebothrombosis, deep vein thrombosis in pregnancy)

^b^ International Classification of Primary Care (ICPC) code U99.01 (renal impairment) or estimated glomerular filtration rate < 60 ml/min per 1.73 m^2^

^c^ Five most prevalent malignancies in the Netherlands (apart from skin cancer): breast cancer, prostate cancer, colon cancer, lung cancer and haematological cancer

^d^ Posttraumatic extradural/subdural/intracerebral haemorrhage, haemoptysis, epistaxis, haematemesis, melaena, haematochezia, haematuria, menorrhagia, postpartum haemorrhage

^e^ Multivariable analyses with stepwise backward elimination (eliminated if *p*-value ≥ 0.05) and with age and CHA_2_DS_2_-VASc score as continuous instead of dichotomous variables

*VKA* vitamin K antagonist, *NOAC* non-vitamin K antagonist oral anticoagulant, *CI* confidence interval, *CVA* cerebrovascular accident, *TIA* transient ischemic attack, *COPD* chronic obstructive pulmonary disease

**Table S4C** Patient characteristics probably associated with a VKA versus a NOAC prescription in patients with new-onset atrial fibrillation diagnosed in 2017

| **Univariable analyses** | | | |
| --- | --- | --- | --- |
| **Variable** | **VKA (*N*= 145)** | **NOAC (*N*= 255)** | **Unadjusted  odds ratio (95% CI)** |
| Male sex | 81 (55.9) | 119 (46.7) | 1.45 (0.96-2.18) |
| Age, years | 79 (69-85) | 73 (66-79) | 1.04 (1.02-1.06) |
| Age ≥75 years | 87 (60.0) | 108 (42.4) | 2.04 (1.35-3.09) |
| CHA_2_DS_2_-VASc score | 3 (2-5) | 3 (2-4) | 1.13 (1.00-1.29) |
| CHA_2_DS_2_-VASc score ≥2 | 126 (86.9) | 211 (82.7) | 1.38 (0.77-2.47) |
| Heart failure | 36 (24.8) | 32 (12.5) | 2.30 (1.36-3.90) |
| Hypertension | 72 (49.7) | 145 (56.9) | 0.75 (0.50-1.13) |
| Diabetes mellitus | 36 (24.8) | 50 (19.6) | 1.35 (0.83-2.21) |
| CVA or TIA | 12 (8.3) | 23 (9.0) | 0.91 (0.44-1.89) |
| Vascular disease^a^ | 36 (24.8) | 37 (14.5) | 1.95 (1.17-3.25) |
| Renal impairment^b^ | 38 (26.2) | 47 (18.4) | 1.57 (0.97-2.56) |
| Dementia | 10 (6.9) | 4 (1.6) | 4.65 (1.43-15.10) |
| Asthma or COPD | 26 (17.9) | 54 (21.2) | 0.81 (0.48-1.37) |
| Malignancy^c^ | 15 (10.3) | 36 (14.1) | 0.70 (0.37-1.33) |
| History of bleeding^d^ | 41 (28.3) | 43 (16.9) | 1.94 (1.19-3.17) |
| **Multivariable analysesꜝ** | | | |
| **Variable** | **Adjusted odds ratio (95% CI)** | | |
| Age, years | 1.05 (1.02-1.08) | | |
| CHA_2_DS_2_-VASc score | 0.75 (0.61-.092) | | |
| Heart failure | 2.57 (1.38-4.79) | | |
| Vascular disease^a^ | 2.36 (1.30-4.28) | | |
| Dementia | 3.80 (1.11-13.04) | | |
| History of bleeding^d^ | 1.76 (1.05-2.95) | | |

Data are n (%) or median (interquartile range)

^a^ Coronary artery disease (angina pectoris, acute myocardial infarction, other/chronic ischaemic heart disease) or peripheral vascular (arterial or venous) disease (intermittent claudication, thrombophlebitis/phlebothrombosis, deep vein thrombosis in pregnancy)

^b^ International Classification of Primary Care (ICPC) code U99.01 (renal impairment) or estimated glomerular filtration rate < 60 ml/min per 1.73 m^2^

^c^ Five most prevalent malignancies in the Netherlands (apart from skin cancer): breast cancer, prostate cancer, colon cancer, lung cancer and haematological cancer

^d^ Posttraumatic extradural/subdural/intracerebral haemorrhage, haemoptysis, epistaxis, haematemesis, melaena, haematochezia, haematuria, menorrhagia, postpartum haemorrhage

^e^ Multivariable analyses with stepwise backward elimination (eliminated if *p*-value ≥ 0.05) and with age and CHA_2_DS_2_-VASc score as continuous instead of dichotomous variables

*VKA* vitamin K antagonist, *NOAC* non-vitamin K antagonist oral anticoagulant, *CI* confidence interval, *CVA* cerebrovascular accident, *TIA* transient ischemic attack, *COPD* chronic obstructive pulmonary disease
